# Dental caries experience, tooth surface distribution and associated factors in 6- and 13- year- old school children from Davangere, India

**DOI:** 10.4317/jced.50779

**Published:** 2012-10-01

**Authors:** Sakeenabi Basha, Hiremath S. Swamy

**Affiliations:** 1 MDS, Reader , Department of Preventive and Community Dentistry, College of Dental Sciences, Karnataka, India.; 2 MDS, Dean cum Director, Government dental college and research Institute, Fort, Bangalore, India.

## Abstract

Objectives: The objective of the present study was to investigate the caries experience and patterns in a sample of 6- and 13- year old school children and to estimate the contributing roles of the likely risk indicators. Study design: Data were obtained from 400 (196, 6-year old and 204, 13-year old) school children. A questionnaire was sent to the children parents to measure socioeconomic, socio-demographic, and behavioral variables. Dental caries detection was performed according to the World Health Organization criteria (dmft and DMFT). The mean dmft/DMFT scores were analysed either as a continuous (calculating means and standard deviations) or as a categorical variable (providing proportions). We also created a multivariate logistic regression model. Results: Overall caries prevalence was dmft > 0 = 26.75% (6-years old = 50.51%; 13-years old = 3.92%) and DMFT > 0 = 25.25% (6-years old = 12.75%; 13-years old = 37.25%). Multivariate analysis showed that presence of enamel defects, low socio-economic status, mothers’ educational levels were significantly associated with caries prevalence in both the dentition, and caries in the primary teeth (OR = 4.87) were associated with DMFT > 0. Most commonly affected teeth were lower first molar in permanent dentition and lower second molars in primary dentition. In both the dentition occlusal surfaces were most often affected compared to other surfaces. Conclusions: This study has identified clinical, socio-economic, and behavioral determinants for dental caries in primary and permanent dentition on Indian schoolchildren.

** Key words:**Dental caries experience, socio-economic status, enamel defects, tooth surface distribution.

## Introduction

Dental caries is one of the main oral health problems in both industrialized and increasing in developing countries and it affects 60 to 90% of school aged children and adults (1). In India, children comprise 40% of a rapidly growing population. The prevalence of dental caries varies from 33.7% to 90% in child population and is increasing at an alarming rate (2-5). According to WHO Global Oral Data Bank in 1997 the point prevalence was 81.5% (mean dmft 4.86) among 5-6 year old and 59.6% (mean DMFT 1.87) among 12-13 year old (6). Using multivariate models, reports from around the world have established the relative importance of specific factor to dental caries experience in children. Among these factors, low income (7), deficient oral hygiene, mother’s schooling, and fluorosis (8), various measures of low socio-economic status (9-11), older age (12), prior experience of decay in the primary dentition associated with caries experience in the permanent dentition (13,14), female sex (15), presence of abnormalities, hypoplasia, or enamel defects (16,17), as well as low level of parental education and cariogenic diet all affect caries risk (2,17). Apart from these, the pattern of dental caries varies between the primary to permanent dentition; this difference in caries susceptibility is no doubt related to differences in tooth morphology (18,19). While various descriptive epidemiological studies of dental caries in children have been undertaken in Indian population and no multivariate models were included to ascertain the relative role of identified caries risk indicators, along with this, information on caries prevalence and severity forms the basis for the magnitude and quality of caries prevention programs and treatment needs in a population. Therefore, a continuous need remains to field caries prevalence and severity information. Thus the present study is designed to assess the prevalence of dental caries (Percentage with caries), caries experience in primary and permanent dentition (dmft and DMFT) of 6- and 13- year-old school children in Davangere city, Karnataka state, India, to assess the surface distribution of dental caries, and to assess the contributing roles of the likely risk indicators like gender, parental education, oral hygiene habits, sugar consumption, socio-economic status, enamel defects, plaque score and dental visits on dental caries prevalence in primary and permanent dentition.

## Material and Methods

Ethical aspects: The study was approved by the Research Ethics Committee of the Government Dental College, Bangalore, India. An Informed Consent Form containing information about the clinical examination that would be carried out was obtained from parents prior to the survey.

Sample: The investigation was conducted in the Davangere city, Karnataka state (India), between June and July 2008. The sample of children included was drawn from school children aged 6- and 13-years living in the City. In 2008, among 164 schools (101 private and 63 public schools) in Davangere there were 2250 children aged 6-years and 1947 children aged 13-years. The sample was recruited from children attending private and public schools using two stage random sampling method- with the school as primary sampling unit and individual children, the unit of enquiry. Parents of all children in the selected schools were approached and asked to give written consent. Based on their agreement 400 children (200 boys and 200 girls) aged 6 years old (196 subjects) and 13 years old (204 subjects) were selected to take part in the study.

Questionnaire: All children received a semi-structured questionnaire to be answered by their parents. This questionnaire was aimed at collecting information on sociodemographic and socioeconomic details like: monthly family income, number of people living in the household, parents’ educational level (dichotomized to 0 year of schooling and 1-15 years of schooling), mothers occupation (dichotomized to homemaker and work out of home), and behavioral variables related to child like: sugar consumption (24 hour before, from midnight to midnight), oral hygiene practices (tooth brushing frequency and use of fluoridated tooth paste), and visit to dentist. Sugar consumption was considered present if the children consumed snacks (cookies, candies, chocolate), fruit juice, non-diet or other sugar-containing drinks. It is classified into three categories, according to form of sugar consumed: Liquid form (sugar in beverages/soft drinks/milk with sugar/infant formula with sugar), solid form (chocolates/confectionaries/home made solid sweets/bread and jam/Biscuits), and combined form (both solid and liquid form). Socioeconomic status (SES) was assessed according to Prasad classification (20) using percapita family income; children were classified into one of the three clusters: upper class, middle class and lower class.

Oral examination: Prior to the examination, the dentist participated in the calibration process, which was divided into theoretical discussions on codes and criteria for the study, and practical activities. Calibration was performed with respect to the diagnostic criteria of caries, enamel defect, and plaque scores. There was a significant correlation with Kappa value of 0.96 for dental caries, 0.85 for enamel defects, and 0.83 for plaque score. Examination of children was carried out under natural light using plane mouth mirrors, World Health Organization (WHO) probes, and explorers. The sterilization of instruments was done by autoclave method. No radiographs were taken. The same examiner examined all children. Diagnosis of dental caries (dmft/DMFT) was established according WHO guidelines (21), which were further differentiated in to occlusal/incisal, buccal/facial, lingual, palatal, mesial and distal lesions. Enamel defects (permanent dentition) were recorded using a modified Developmental Defects of Enamel index (DDE) (22). Dental plaque was measured using a modification of the Silness and Loe index (23). In 6 years old, where permanent teeth were not erupted, the respective deciduous teeth were scored (Federation Dentaire International (FDI) tooth numbers-55, 52, 64, 75, 72, and 84) for plaque.

Statistical analysis: Descriptive summary statistics were obtained for all demographic and outcome variables. Difference in proportion was tested using Kruskal-Wallis H and Chi-Square tests. Difference in means was tested using Independent sample *t–test*. Relationships between dental caries (primary and permanent dentition) and other factors were assessed using multivariable logistic regression. Adjusted odds ratios (ORs) and their 95 percent confidence intervals (CI) were calculated. In evaluating the association, we adjusted for the following confounders: gender, sugary product consumption (dichotomized to liquid form and both solid and liquid form), oral hygiene habits (tooth brushing frequency and fluoridated dentifrice), school type (government/private), mother’s education, father’s education, mother’s occupation, visit to dentist, plaque score (dichotomized to good (excellent and good scores combined), and fair (fair and poor scores combined)), enamel defects (dichotomized to with and without defects) and SES. Analysis was performed using Statistical Package for Social Science version 17 (SPSS INC Chicago link). All statistical tests were two-sided, and the significance level was set at P < 0.05.

## Results

Of the 400 school children included in the study, 196 (49%) were 6-year-old and 204 (51%) were 13-year-old. 182 (45.5%) children were from government schools and 218 (54.5%) from private schools. Caries prevalence (overall) was 26.75% in primary dentition (dmft>0) and 25.25% in permanent dentition (DMFT > 0). Caries prevalence in relation to various risk indicators is given in  Table 1. There was no significant difference (p>0.05) in caries prevalence in relation to gender, type of school, tooth brushing frequency and mother’s occupation. However, there was significant difference (p<0.05) in caries prevalence in relation to age, SES, use of fluoridated dentifrice, sugar consumption, parental education, plaque score, enamel defects and dental visits. The mean dmft and DMFT score for 6-year-old was 3.20±4.25 and 0.23±0.7 respectively. In both the dentition mean dt (3.18±4.21) and DT (0.23±0.7) scores predominated over filled component. In 13-year-old, mean DMFT score was 1.40±2.35 (DT = 1.32±2.30). No significant gender differences were observed (p>0.05) for caries indices.

**Table 1:**
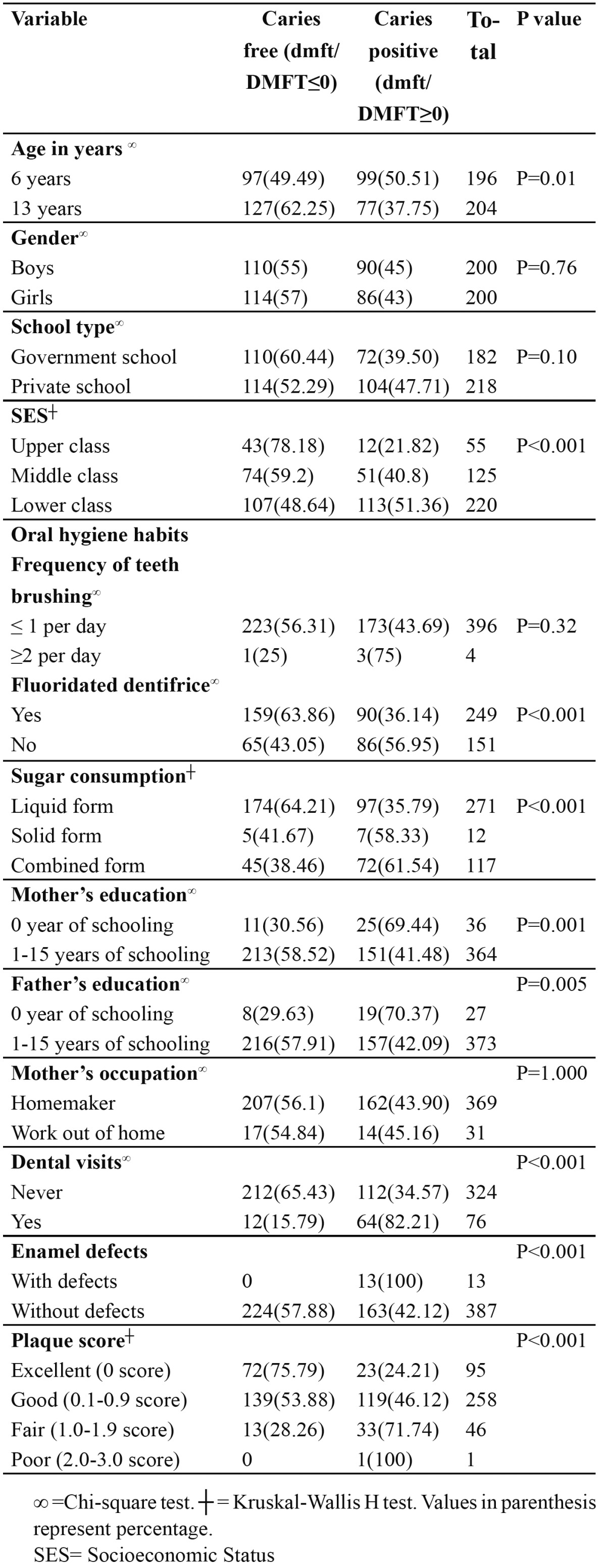
Caries prevalence in relation to age, gender, school type, SES, oral hygiene habits, sugar consumption, parental education, mother’s occupation, visit to dentist, enamel defect and plaque score.

In primary dentition, most affected teeth were mandibular second molars (23.51%), followed by maxillary second molars (16.46%), mandibular first molars (15.91%), maxillary first molars (13.74%), and maxillary anteriors (11.93%). Mandibular anteriors were least affected (3.98%). In permanent dentition mandibular first molars (35.58%) were most frequently affected with decay followed by maxillary first molars (25.96%), mandibular second molars (18.91%), and maxillary second molars (13.78%).

In both the dentition, it was frequent to find decay on occlusal surfaces and rare in lingual /palatal surfaces ( Table 2). The result of multivariate analysis is given in  Table 3 for primary dentition. Caries experience was associated with sugar consumption (OR= 2.31; 95% CI = 1.72-3.10), dental visits (OR= 2.10; 95% CI = 1.32-2.90), presence of enamel defects (OR= 2.69; 95% CI = 1.28-3.10), and plaque score (OR= 1.38; 95% CI = 1.01-1.98). Children from low SES had an OR 1.33 times higher likelihood of having caries than children from medium and upper SES (95% CI = 1.02-1.89).

**Table 2:**
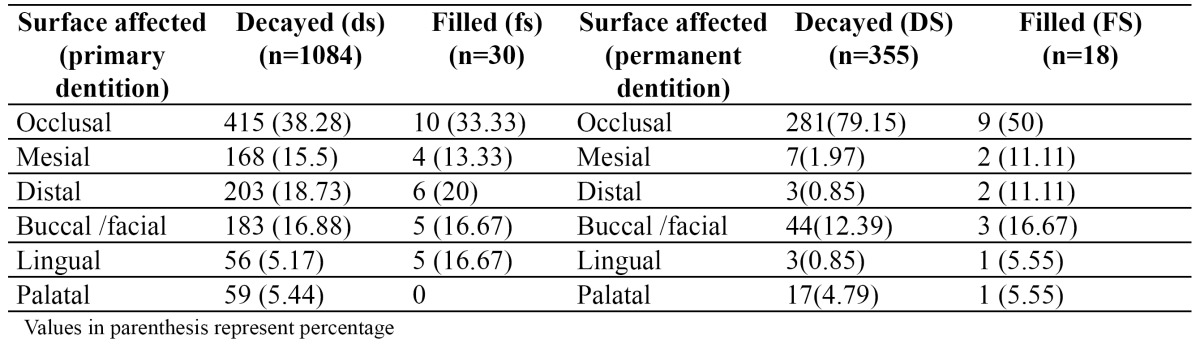
Caries experience in primary and permanent dentition according to surface affected.

**Table 3:**
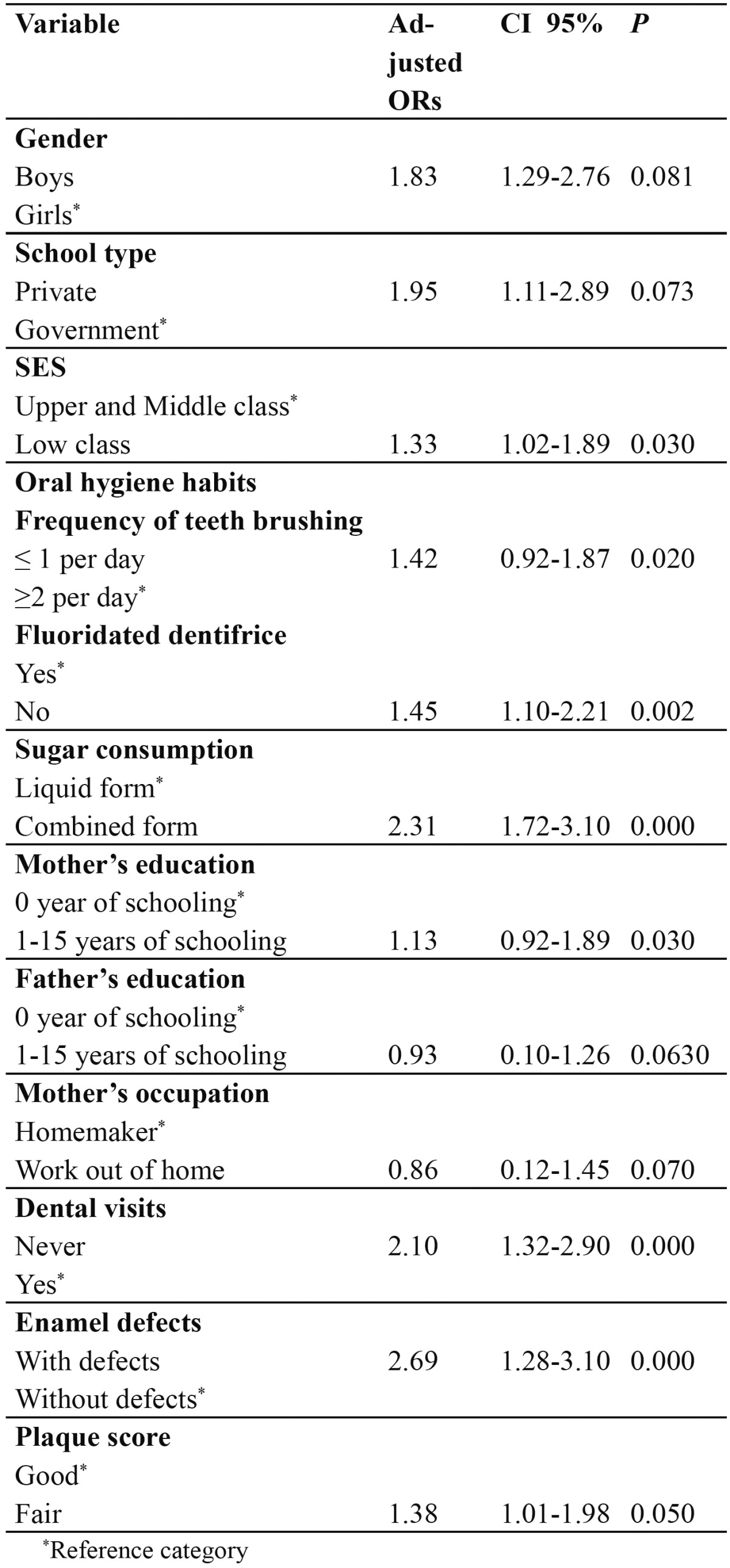
Logistic regression multivariate model for caries on primary dentition (n = 107), Dependent variable, dmft, was dichotomized as dmft = 0 versus dmft > 0.

The result of multivariate analysis for permanent dentition is given in  Table 4. Children with caries in their primary teeth had a strong association (OR= 4.87; 95% CI = 2.33-6.87) with caries experience. The children with fair plaque score had an OR 2.45 times higher likelihood of having caries than children with good plaque score (95% CI = 1.12-4.32). The presence of enamel defects (OR= 3.92; 95% CI = 2.13-4.89) was also positively associated with caries.

**Table 4:**
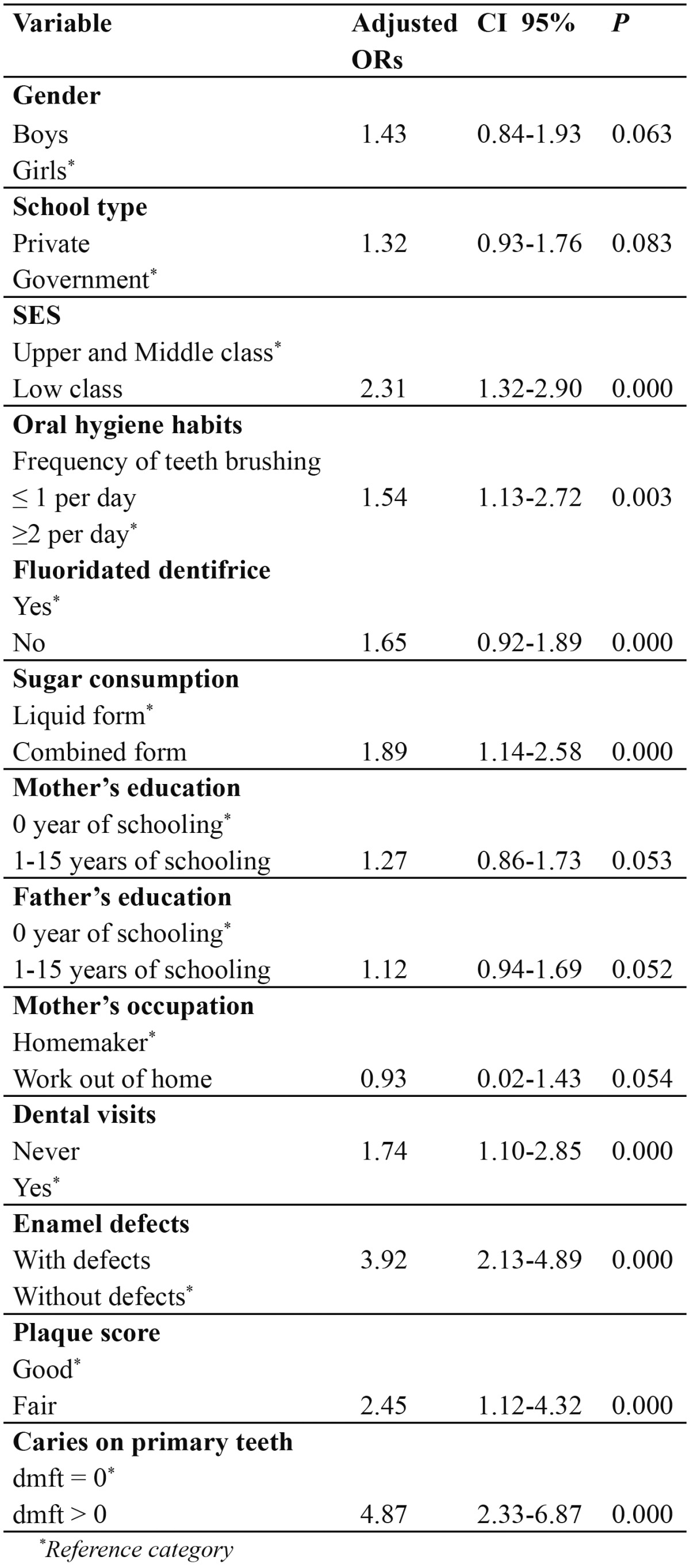
Logistic regression multivariate model for caries on permanent dentition (n = 101), Dependent variable, DMFT, was dichotomized as DMFT = 0 versus DMFT > 0.

## Discussion

The present study provides information on dental caries status and associated factors, with detailed information on teeth and surface distribution of caries in a representative sample (n=400) of 6- and 13-year-old school children in Davangere City, Karnataka state, India. The available studies on dental caries indicate that caries is a public health problem because of its high prevalence and incidence (1,4,7,9,12). While the prevalence of caries in our study was slightly lower than figures commonly reported in other epidemiological studies in India (both caries prevalence and dmft and DMFT scores) (3-5), bringing our findings closer to the WHO/FDI goals for 2000 in children ages 6 and 12 years (24). In the present study, prevalence of caries was higher in 6 years age group when compared to 13 year age, and the difference was statistically significant. This could be due to the increase in age; there is increased awareness of oral hygiene. Permanent teeth are also more resistant to caries process than primary teeth (9). There was no significant difference in caries prevalence between gender, this results coincide with the previous Indian studies (2,4,5). In the present study, decayed component of caries experience predominated over filled component, both in primary and permanent dentition. This high proportion of unrestored teeth is consistent with findings from other developing and underdeveloped countries (25,26). Because dental services in Karnataka are not free, children have to seek care from private dentists, which are considered expensive. Furthermore, a low perception of need for treatment and the low priority placed on oral healthcare compared with other needs could be reason for not restoring teeth (2,26). A childs economic back-ground has also been shown to influence the probability of seeking dental care.

Eighty one percent of children in the present study had never visited a dentist, compared with 11% in Jordan (27) and 42% in China (28). Possible explanation for this low dental visit may be that there is no tradition of visiting a dentist and only children who have problems with their teeth seek dental care. In the present study caries prevalence was higher among children who never visited a dentist than those who visited dentists. This finding is contradictory to the previous studies (2,27,28) which showed high caries prevalence among children who visited dentists. The reason coated was that the dental visits by children were symptom-oriented.

The caries preventive effect of fluoride toothpaste has been well documented (29). In the present study 62.25% of children used fluoridated dentifrice, and this was significantly associated with caries prevalence both in primary and permanent dentition. Both, mother’s and father’s education significantly associated with caries prevalence in the present study. This is in accordance with the study conducted by David et al. in India (2).

A low level of restorative dental care (only few fillings) in the present study group makes them particularly suitable for the study of caries patterns, since the distribution of the lesions was not modified by treatment decisions of the dentist. Caries lesions were not evenly distributed among different tooth types. In permanent dentition, lower first molar was more often found to be decayed than the upper first molars, followed by lower and upper second permanent molars. Occlusal surfaces most frequently affected with decay compared to other surfaces. These findings are in agreement with an earlier report on caries attack patterns in children of the same age in US (19). In primary dentition, lower second molars most frequently affected with decay, compared to upper second molars. Primary first molars in both the arches are less susceptible to caries than the primary second molars, even though the former erupts at an earlier date. This suggests that in primary dentition, the second molar is the tooth with highest caries experience. This difference in individual tooth susceptibility is due to the fissure topography of molars. The pits and fissures in second primary molars are deeper and less completely coalesced (18). It is also evident that the sequence of caries attack follows a specific pattern in primary dentition: mandibular molars, maxillary molars and maxillary anterior teeth were predominantly affected by caries, whereas the mandibular anterior teeth were least affected. This is similar to the caries pattern described by Saravanan et al (18).

In the present investigation, enamel defects were a strong predictor for dental caries on the primary and permanent dentitions, after adjusting for other variables. Other authors (16,17) have observed this association between hypomineralized enamel (presumably it can help bacterial colonization) and dental caries. The significant relationship between oral hygiene habits and dental caries in permanent and primary dentitions had been reported previously (8,17). Mother’s education level was significantly associated with caries prevalence both in primary and permanent dentition, as observed by other authors (8). In general, the consistent association between more disadvantaged socio-economic background and increased caries experience in other countries was confirmed (9-11). The most important aspect of such confirmatory association between DMFT and dmft indices and SES is that this link remained even after controlling for other variables. Our findings suggest that the caries experience in primary teeth is a major predictor for caries in permanent dentition, thus substantiating longitudinal reports from other countries (13,14,). This variable, as well as others evaluated in the present analysis, could be useful in identifying subjects with a high caries risk (14,30) following today’s standards of care. This study had certain limitations that call for a cautious interpretation of the results. A cross-sectional data measures cause and effect at the same point in time, introducing the problem of temporal ambiguity and an inability to establish causal relationships. Furthermore, the fact that questionnaires were used directed at parents/guardians to collect information could be introducing some degree of recall bias. No radiographs were used in this study.

To conclusion, dental caries status for the sample of Indian children in this study was favorable compared with previous studies, i.e. it being closer to the goals proposed by the WHO/FDI for 2000. The distribution of carious lesions both in primary and permanent dentition follows specific patterns. The study has identified clinical, socio-economic and behavioral determinants for dental caries in both dentitions on Indian schoolchildren. Such positive trends may be emphasized through preventive programs that meet population treatment needs by targeting resources through objective risk assessment, and by ameliorating dissimilar disease experiences between social classes. Epidemiological data can thus be used for designing programs aimed at improving oral health services in this community. A longitudinal study needs to be undertaken in this population, to confirm these results.
